# Anti-α-Amino-3-Hydroxy-5-Methyl-4-Isoxazolepropionic Acid (AMPA) Receptor Antibody Encephalitis in an Immunosuppressed Patient With Myasthenia Gravis Post-thymoma Treatment

**DOI:** 10.7759/cureus.63239

**Published:** 2024-06-26

**Authors:** Suyuan Tan, Heitor Frade, Kian S Abdul-Baki, Samantha De Gannes, Puneet Singh, Ernesto Lopez Valencia, Anand Kumar, Aimalohi Esechie, Chilvana Patel

**Affiliations:** 1 Internal Medicine, The University of Texas Medical Branch, Galveston, USA; 2 Neurology, The University of Texas Medical Branch, Galveston, USA; 3 Critical Care Medicine, The University of Texas Medical Branch, Galveston, USA; 4 Anesthesiology and Critical Care Medicine, Massachusetts General Hospital, Harvard Medical School, Boston, USA

**Keywords:** encephalopathy, nmda, thymoma, autoimmune, receptor, encephalitis, ampa

## Abstract

Anti-α-amino-3-hydroxy-5-methyl-4-isoxazolepropionic acid (AMPA) receptor encephalitis is a rare form of autoimmune encephalitis. We report a case of a patient with myasthenia gravis post-thymoma treatment who presented with deteriorating mental status. She was found to have positive AMPA receptor titers in her cerebrospinal fluid and subsequently attained full recovery. Of the limited number of cases that were documented, we report the only case of a patient who was previously immunocompromised to develop the condition. Even though autoimmune encephalitis is an emerging condition, its early differentiation from other causes of encephalitis is crucial in the prognosis of the patient.

## Introduction

Paraneoplastic neurological syndromes, although relatively uncommon, have become increasingly prevalent over the last two decades and affect nearly 1 in 300 cancer patients. Among its high-risk phenotypes is limbic encephalitis, a condition characterized by rapidly progressive short-term memory loss, seizures, hyperkinetic movement, and psychiatric manifestations [1,2].

Anti-α-amino-3-hydroxy-5-methyl-4-isoxazolepropionic acid (AMPA) encephalitis is a rare form of paraneoplastic encephalitides, which have been associated with onconeural antibodies such as anti-Hu, anti-Ma2, and anti-N-methyl-D-aspartate (anti-NMDA). Although AMPA receptors are predominant in the hippocampus, they are also widely expressed in the subiculum, cerebellum, striatum, and cerebral cortex. This widespread expression suggests its clinical presentation could extend beyond that of limbic encephalitides [3].

Patients with anti-AMPA encephalitis classically present with limbic symptoms, including behavior, mood, and memory changes, but patients can also present with other neurological symptoms such as seizures, somnolence, and hallucinations [4]. The malignancies associated with anti-AMPA encephalitis are most commonly small-cell lung carcinoma, thymoma, and breast carcinoma [3]. As of writing this case report, there is not yet a standardized treatment for anti-AMPA encephalitis. Most documented cases were treated with some combination of steroids, plasmapheresis, and intravenous immunoglobulin (IVIG), with mixed results. The cases with the most favorable prognosis are those in which the underlying cancer is treated.

We present a case of a 43-year-old female diagnosed with anti-AMPA receptor encephalitis in the setting of immunosuppression. It is important to note that our patient’s immunosuppressed status makes our case unique as compared to previously reported cases.

## Case presentation

A 43-year-old woman with myasthenia gravis (acetylcholine receptor binding antibody titer of 76 nmol/L, Myasthenia Gravis Activities of Daily Living score 0) on pyridostigmine and mycophenolate mofetil, with a history of thymoma treated with thymectomy and radiation therapy one year prior, acutely developed rapidly deteriorating mental status. She initially presented to urgent care, where her symptoms were attributed to a sinus infection and possible pneumonia. Head computerized tomography (CT) was unremarkable.

She was discharged home with steroids and antibiotics for her infection but became increasingly more disoriented the following day. The patient was subsequently seen in the emergency department, where she was admitted to the medical intensive care unit (ICU) for rapidly deteriorating mentation and concerns for airway protection.

**Figure 1:**
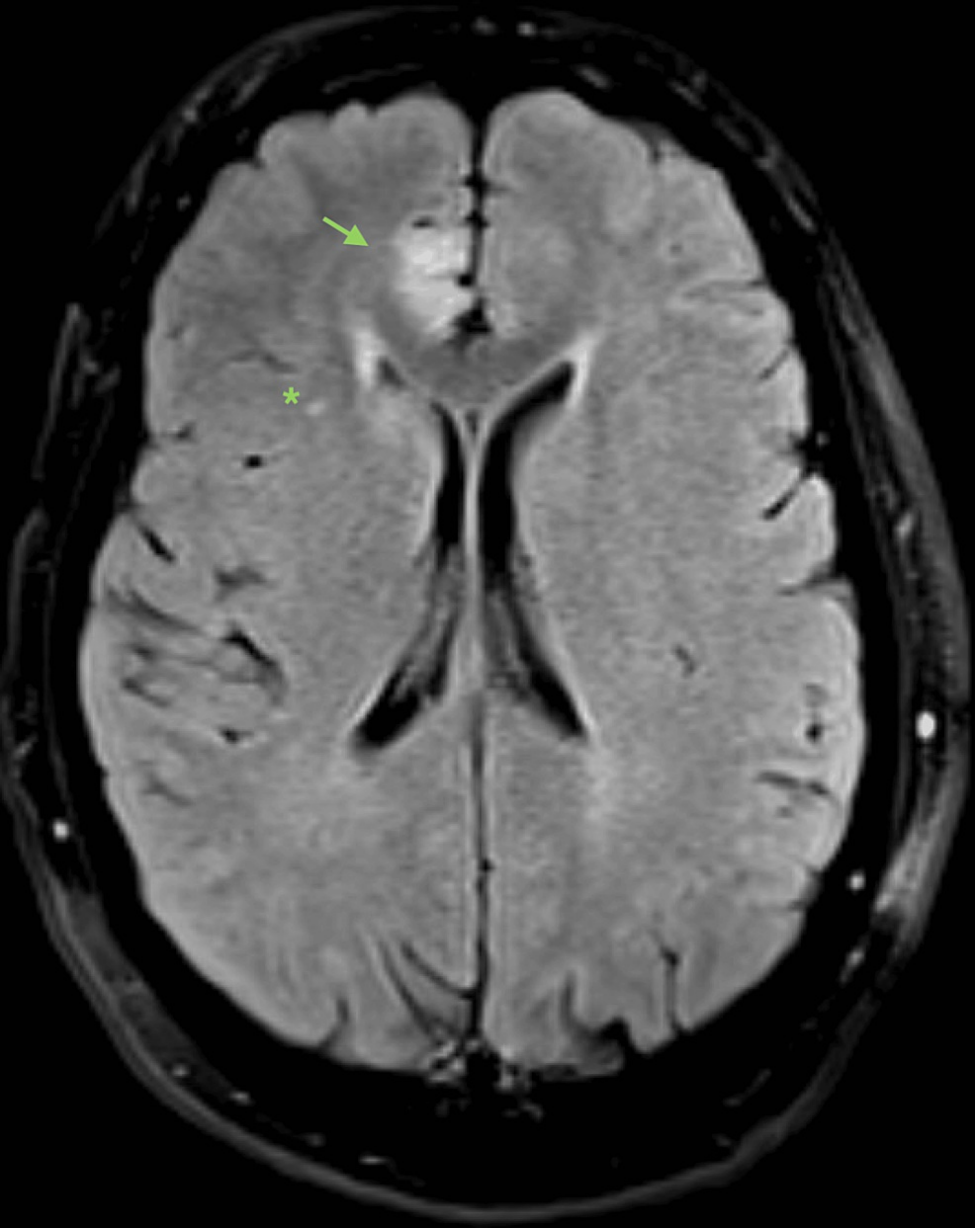
Fluid-attenuated inversion recovery magnetic resonance imaging (FLAIR MRI) from admission day 2, axial section, showing a right mesiofrontal hyperintense focus (arrow) and a right subcortical punctate hyperintense focus (*).

**Figure 2:**
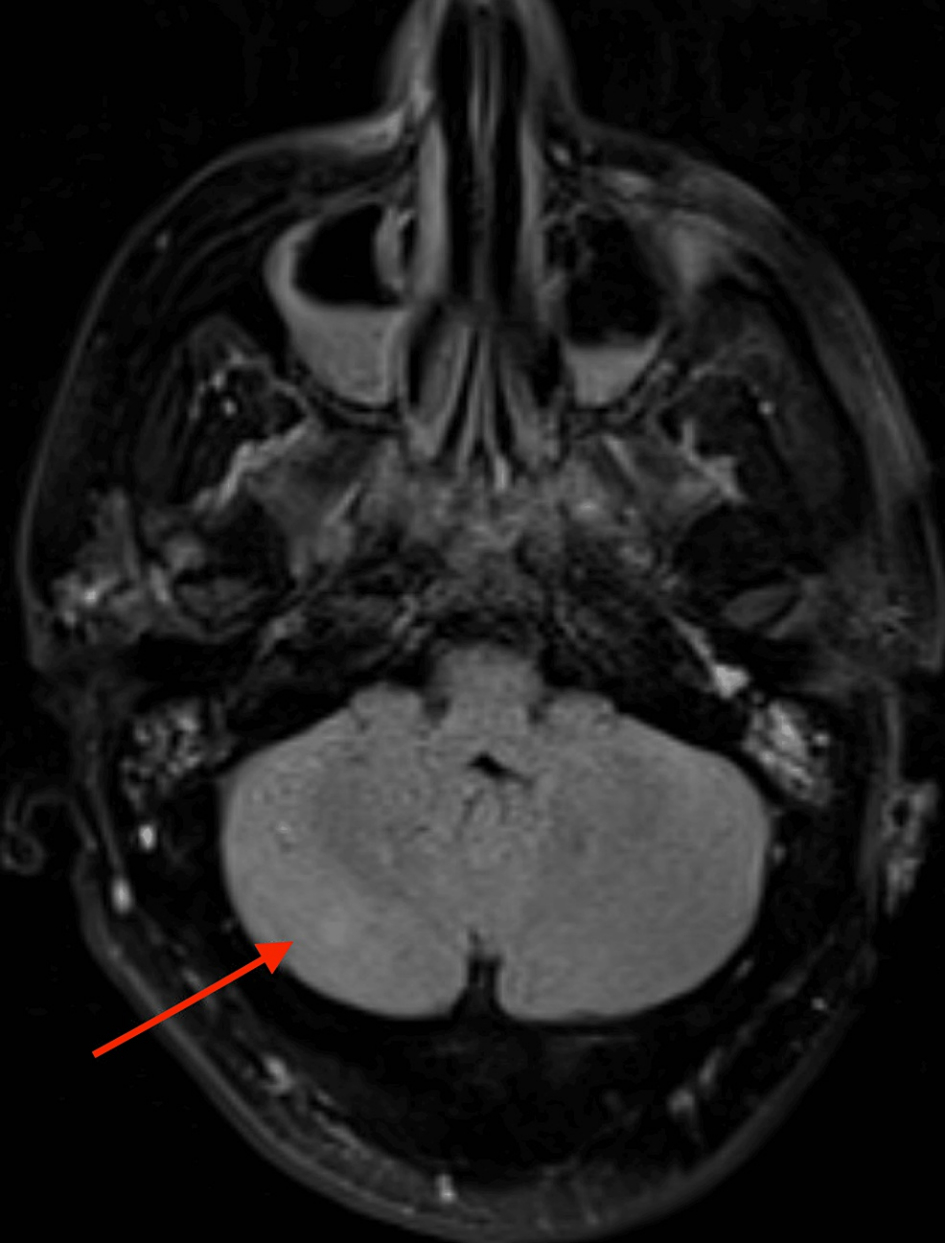
The same fluid-attenuated inversion recovery magnetic resonance imaging (FLAIR MRI) showing an infratentorial lesion in the right cerebellar hemisphere (red arrow).

On admission, the patient was alert and oriented only to a person. She was somnolent but arousable to voice and was able to follow commands. However, she did not have meaningful conversations. Brain stem reflexes as well as sensation and strength were intact throughout. Hoffman and Babinski's reflexes were absent. Ankle, patellar, bicep, and tricep reflexes were 2+ bilaterally. The patient did not exhibit nystagmus, tremors, or focal neurological deficits. The day following admission, she was noted to have a new onset right facial droop, raising concern for a cerebrovascular accident. Her mental status continued to deteriorate, and she was intubated for airway protection. Magnetic resonance imaging (MRI) of the brain with and without contrast showed multifocal supra- and infratentorial hyperintensities suggestive of meningitis/encephalitis (Figures 1-2). On interval examination, she withdrew from pain stimuli in all four extremities, had symmetric deep tendon reflexes, and showed an absent Babinski sign, but no nuchal rigidity was appreciated. Lumbar puncture (LP) was performed, and cerebrospinal fluid (CSF) showed a protein of 85, a white blood count (WBC) of 60 (with lymphocytic predominance), a red blood count (RBC) of 4,000, and a glucose of 67. Herpes simplex virus (HSV)-1 IgG screen was positive, but the HSV nucleic acid amplification test (NAAT) was negative. The LP findings, in addition to a positive HSV-1 IgG, raised suspicion for HSV encephalitis. The infectious disease specialists were consulted and recommended a 21-day course of acyclovir. 

Repeat LP one week later showed similar CSF results but with a down-trending WBC and RBC of 11 and 11, respectively, and an opening pressure at 43 cm H_2_O. HSV NAAT was not repeated on this LP attempt. The serum autoimmune encephalitis panel revealed anti-thyroid peroxidase antibody levels of 267.1 WHO units (reference range: 0-100.0 WHO units), despite thyroid stimulating hormone and free T4 levels being within normal limits. The send-out CSF autoimmune panel returned with notable findings of high AMPA receptor (AMPAr) titers (1:160) and negative results for NMDA receptor (NMDAr), GABA-B receptor, GAD, or CASPR2. Several routine and continuous electroencephalograms were performed, revealing diffuse slowing and frontal intermittent rhythmic delta activity, but no electrographic seizures were observed. A five-day course of IVIG was completed for empiric treatment, followed by five days of pulse dose steroids, with minimal interval improvement. The patient was subsequently started on plasma exchange (PLEX) seven days after IVIG completion. However, the neurological exam remained unchanged. The patient was then transferred from the medical ICU to the neurological ICU for further management. Given the association of anti-AMPA encephalitis with paraneoplastic syndromes and malignancies of the thymus, lung, breast, and ovaries, gynecologists were consulted. MRI pelvis showed uterine fibroids but no adnexal masses or underlying pelvic malignancy. Her hospital course was complicated by tracheostomy and percutaneous endoscopic gastrostomy tube placement in addition to ventilator-associated pneumonia for which a course of broad-spectrum antibiotics was completed. The patient was then discharged to a long-term acute care hospital on suppressive glucocorticoids where she spent roughly one month and was subsequently admitted to an inpatient rehabilitation facility for two weeks with significant clinical improvement. She has since been discharged home. Family members reported that the patient was not able to remember her prolonged hospitalization but otherwise did not suffer any long-term neurological deficits. At the time of writing this case report, she had regained her strength and sensation, could ambulate independently, and could speak again. She continues to attend neurorehabilitation therapy sessions on an outpatient basis.

## Discussion

Encephalitis is a neurologic emergency that requires rapid diagnosis and initiation of treatment. Though autoimmune encephalitis is an emerging entity primarily attributable to the increasing recognition of NMDA receptor encephalitis, there is a spectrum of autoimmune disorders that present with various encephalitis syndromes that go underdiagnosed and are, therefore, undertreated.

Clinical presentation 

Anti-AMPA encephalitis, within itself, can present with limbic encephalitis, which includes a wide array of symptoms, including short-term memory deficits, behavioral disturbances, seizures, and psychomotor agitation [3]. Acute psychosis has also been documented in the literature as a presenting symptom [4]. 

Typical associated tumors

Our patient had a history of thymic carcinoma that was previously diagnosed during the evaluation of myasthenia gravis. Prior case series have reported links between anti-AMPA encephalitis and tumors of the thymus, breast, lung, and ovaries [3,4].

There were few cases, however, in which no underlying neoplasm was found despite extensive evaluation, and there has been at least one documented case that developed despite prior successful treatment of a thymic carcinoma. In our patient, her thymic carcinoma was treated previously with resection followed by multiple rounds of radiation therapy due to small perivascular remnants of the tumor. During admission, we pursued extensive imaging workup for malignancy, including CT thorax, abdomen, and neck, and an MRI of the pelvis. All showed no recurrence of thymic cancer. It has been suggested that such presentations may be related to autoimmunity triggered by prior expression of AMPA receptor subunits that may persist after tumor treatment [3].

Association with other autoimmune conditions

Lai et al. reported five of 10 other cases in which comorbid autoimmune diseases have been associated with anti-AMPA encephalitis. Other autoimmune conditions associated with anti-AMPA encephalitis include type 1 diabetes mellitus, Raynaud’s disease, as well as Stiff person syndrome [3]. Our case is one of the few reported anti-AMPA encephalitides associated with thymomatous myasthenia gravis, with the first reported by Li et al. in 2015 [5]. Schäfer et al. also presented a case of combined autoimmune encephalitis in the setting of seronegative myasthenia gravis [6]. While these studies show that autoimmune encephalitides are associated with other autoimmune conditions, our case is unique in that the patient was on an adequate immunosuppressive regimen for her myasthenia gravis, creating a dilemma in reaching a final diagnosis.

Also in our case, the patient was found to have elevated thyroperoxidase (TPO) antibodies. Although Hashimoto encephalopathy was considered, it was later deemed unlikely given the lack of response to steroids, IVIG, or PLEX, with the latter two treatments being particularly effective in steroid-resistant cases [7,8].

Imaging findings and diagnostic labs

Usual MRI findings of anti-AMPA receptor encephalitis include increased flair signal abnormalities in the medial temporal lobes [4]. Our case showed multifocal lesions involving both the cortex (particularly the parasagittal frontal lobes) and the subcortex (Figures 1-2). Though these findings can confirm an encephalitis syndrome, they cannot differentiate between autoimmune or infectious etiologies. Similarly, CSF analysis can demonstrate the pleocytosis that is often associated with other sterile encephalitis syndromes. In our case, eventual confirmation of the diagnosis did come from polymerase chain reaction (PCR) testing of CSF, but the antibody-mediated encephalitis PCR testing was sent to an outside lab. Therefore, establishing the diagnosis was delayed because we did not receive the results until two weeks following admission. This delay prompted us to initiate empiric treatment without having a definitive diagnosis, including treatment for HSV encephalitis despite a negative HSV PCR and only a positive HSV IgG, which is not specific for active infection. Although it may not be cost-effective to send autoimmune CSF testing on every patient presenting with encephalitis, only doing so when the usual standard of care has failed could delay making the diagnosis and initiating treatment. Despite the severity of symptoms and alarming features associated with anti-AMPA encephalitis, early recognition and treatment can often result in nearly full recovery.

## Conclusions

To our knowledge, our case is the only reported patient with AMPA-receptor encephalitis in the setting of immunosuppression. This was a diagnostic challenge for many reasons. HSV encephalitis remained at the top of the differential diagnosis, given the patient’s immunosuppression and positive HSV IgG. This anchoring bias resulted in a hesitancy to consider alternative diagnoses. A further confounding piece of the patient’s medical history was her thymoma resection. As mentioned previously, there are very few reported cases describing AMPA-receptor encephalitis recurrence after malignancy resection. We were hesitant to consider the possibility of a paraneoplastic encephalitic syndrome developing after the treatment of an underlying tumor. Furthermore, as mentioned previously, there was a delay in making the diagnosis due to send-out lab testing, which also contributed to the diagnostic dilemma. Ultimately, our patient had a delayed but nearly full recovery after discharge from our service following a combination of IVIG, steroids, and PLEX therapy. Our case aims to shed light on this rare and elusive diagnosis and emphasizes the importance of considering paraneoplastic encephalitis in patients presenting with acute neurological symptoms, particularly in those who are immunosuppressed or have a history of treated malignancy.
